# Supplementary material for: Methylglyoxal Decoration of Glutenin during Heat Processing Could Alleviate the Resulting Allergic Reaction in Mice
Source: Nutrients. 2020 Sep 17;12(9):2844. doi: 10.3390/nu12092844 (PMC7551842; doi:10.3390/nu12092844)
Supplement: Supplementary file 1 [file nutrients-12-02844-s001.pdf]

Supplementary Data

Methylglyoxal decoration of glutenin during heat processing could alleviate the resulted allergic reaction by stimulating Treg cell differentiation in mice

Yaya Wang[1], Xiang Li[1], Sihao Wu[1], Lu Dong[1], Yaozhong Hu[1], Yan Zhang[1],

Junping Wang[2], Shuo Wang [1*]

Experimental design

Mice were intraperitoneally sensitized with 10μg of glutenin absorbed on aluminum hydroxide (Sigma-Aldrich, Saint Quentin Fallavier, France) on days 0, 7, 14,21 and 28. Challenge was performed by intragastric administration of 20 mg of glutenin on 35 days.

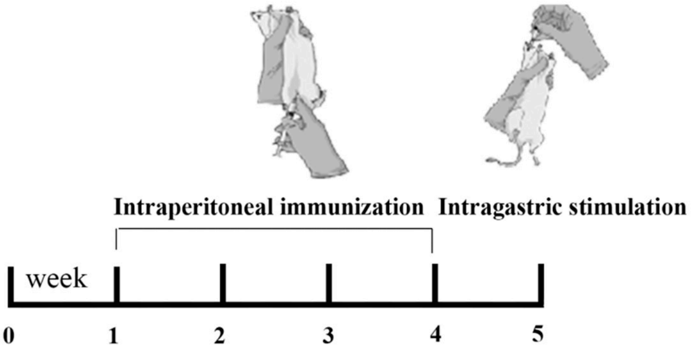

Figure S1. Schematic diagram of mouse sensitization model.

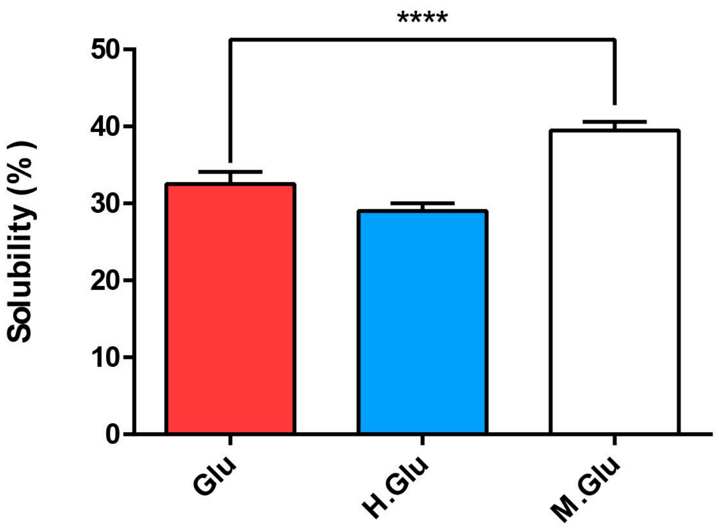

Fig. S2. Solubility of native glutenin, heated glutenin, and heated MGO-glutenin samples.
